# American Versus European Inspection1Read before the Missouri Valley Veterinary Association.

**Published:** 1900-03

**Authors:** A. T. Peters

**Affiliations:** Lincoln, Nebraska


### AMERICAN *versus* EUROPEAN INSPECTION.[1]

BY A. T. PETERS, M.R.C.V.S.,
LINCOLN, NEBRASKA.

I THANK you for the kind invitation to speak to you on such a broad and interesting subject as that of meat-inspection, and will, therefore, try to say a few words on this vast subject. There is probably no one more qualified to point out its virtues and its faults than this honorable body gathered here to-night; but if you will bear with me I will compare our system of meat-inspection with that of Europe, and especially Germany, which I happen to know something about, and also state the rapid improvement that has been made in recent years in this country in this line.

Meat-inspection in this country has only a very recent history, but one that we can be proud of. In the older countries of Europe meat-inspection has been in vogue for a long time, but it is only in the last fifteen years that it has received a thorough reorganization all over the world. In reality it first took a steady boom in the old country when pathology became established on a more thorough basis, and with our increased knowledge of contagious and infectious diseases it became more defined; for instance, the disease, tuberculosis, in years gone by was diagnosed as many different diseases, and it took many years to clearly demonstrate its infectiousness. Any one who is especially interested in meat-inspection, and especially in pathology, would be surprised to read the history of tuberculosis and to see in what state of affairs pathology was before this disease was thoroughly demonstrated. We owe this to Robert Koch, who demonstrated the infection beyond a doubt. But before Koch it was our veterinary profession that pointed out very clearly the infectiousness of tuberculosis. It took a man with a great deal of courage and personality to defy the medical men of that age and to draw attention to their mistaken methods of investigation. This man was Professor Gerlach, who is well known to all of us. He was the first one to draw attention to the fact that tuberculosis is a distinct infectious disease; and later Dr. Robert Koch proved beyond the shadow of a doubt that tuberculosis is produced by a bacterium; and after thoroughly demonstrating this on many hundreds of animals, it still took time for such men as Virchow to classify the many diseases which were

[1] Read before the Missouri Valley Veterinary Association.

confounded with tuberculosis; and during this time the rapid strides that were made in the perfection of the microscope and in the technique of bacteriology aided our profession to thoroughly become guardian of the public health.

The inspection of meat is such a large subject and one of such great importance that any one who is connected with the performance of this duty may certainly be called a benefactor to the community. I will admit that it has not been established very long, but when we consider how short a period meat-inspection has been in vogue in this country the success is gratifying; for it is only a short time ago that positions now held by the best qualified men were in the hands of politicians who were ridiculed by all scientific men. It is surprising to those who have observed the short space of time in which inspection of meat has outgrown the prejudice of the people, for we can certainly say that since the civil service has been established these very important positions are held by the best men in the country; and probably there is no branch of the veterinary profession which has made such rapid progress and which has been so appreciated by the people as this one branch.

It would certainly be interesting to many people to know how this large organization of men is systematically working and accomplishing so much good to the country, both from an economical and sanitary stand-point. I have omitted the statistical data, which can be found in the *Year-book of the United States Department of Agriculture* for 1898, which exhibits interesting data on this subject. I can safely say, without any malice toward the European inspectors, that they are not as well organized, nor could they perform the vast amount of work that is being performed by our inspectors in this country. We read to-day a great deal about meat-inspection, and especially from European sources, but allow me to tell you that it must be admitted—and would be privately admitted by the best men in Europe—that our corps of inspection is as efficient and our system of inspection as perfect as any in the world. With the system that prevails in Europe we could never in this busy country of ours complete the examination of the hundreds of thousands of animals that are being inspected. We must certainly admit that they have some very prominent men in this branch of work—such men as Ostertag, Simmons, Long, Fischoeder, and ever so many others. In Europe each province, district, and locality has supervision over its own inspectors, and the chief is not directly responsible for his subordinates. The entire corps of inspectors—no matter if they are engaged in an abattoir in the southern part of Germany or in the northern part of Prussia—are only responsible to their community and not responsible to the Federal Government. For this reason they have special periodicals on municipal meat-inspection which are wholly supported by inspectors of the different abattoirs, who contribute papers of various kinds. This is not the case in this country. The meat-inspectors are responsible to the chiefs of the Federal Government, and for this reason they have not published as many scientific papers on the diseases found on the floors of the abattoirs. These things probably could be remedied by a journal in the English language, similar to that published by Professor Ostertag, giving short descriptions of some of the rare cases seen on the floors of the abattoirs, in this way keeping the interest of the inspectors alive. But I suppose this is not at all practical under the present system ; but some method should be adopted by the Federal Government of stimulating interest in writing up the rare cases found by the inspectors ; and I believe there is no better way than to have either a separate organ for this purpose or space in some of the journals that are now in existence, exclusively for the use of meat-inspectors, and in which the social feature as well as the professional may be represented.

In conclusion I may say that the most severe test that meat-inspection has been put to was probably that during our late war with Spain, when the newspapers contained so many editorials on this subject. All that can be said is that it stood the test, and that those who were inclined to ridicule our methods of inspection must admit that it is as good as any country can produce ; and in closing I wish to say to those members who are engaged in this profession that with their zealous efforts we can bring it up to a standard that will be far superior to that of any other country.

